# Thermal insulation and mechanical performance of sustainable rammed earth walls incorporating construction and demolition waste and calcium oxide

**DOI:** 10.1038/s41598-025-30472-w

**Published:** 2025-12-15

**Authors:** Waleed Fouad, Osama Youssf, Reda Y. M. Allam, Ahmed M. Tahwia

**Affiliations:** 1 Engineering Technology and Environmental Management, Mansoura University, Mansoura, Egypt; 2 Structural Engineering Department, Mansoura University, Mansoura, Egypt; 3 Civil Engineering Department, Horus University-Egypt, New-Damietta, Egypt

**Keywords:** Rammed earth, Thermo-mechanical performance, Thermal insulation, Time lag, Decrement factor, Low-carbon buildings, Climate-responsive design, Energy science and technology, Engineering, Environmental sciences, Materials science

## Abstract

The construction industry must reduce its environmental footprint and use sustainable materials with low energy and carbon emissions. Conventional masonry and concrete are reliable, durable, and widely used construction materials, but they use up natural resources and produce a considerable amount of CO_2_ emissions. Rammed earth (RE) is a sustainable material and an environmentally friendly construction method that is less energy intensive and exhibits good thermal performance; however, its strength is a limitation for larger structural projects. To address these challenges, this study presents an experimental evaluation of the thermo-mechanical, environmental, and economic performance of stabilized rammed earth (RE) walls incorporating construction and demolition waste (CDW) and calcium oxide (CaO) as sustainable stabilizing additives. This research aims to enhance the structural integrity, thermal insulation, and sustainability of RE systems by partially replacing natural soil with CDW (10–30%) and CaO (2–6%). Seven mix designs were designed and tested for compaction properties, unconfined compressive strength (UCS), thermal conductivity, embodied energy, CO_2_ emissions, and thermal behavior under simulated hot climate conditions with varying relative humidity. The optimal mixture, CDW30–C2 (30% CDW and 2% CaO), achieved a peak UCS of 9.3 MPa at 28 days, the lowest thermal conductivity (0.88 W/m·K), moderate embodied energy (705.27 MJ/m^3^), and reduced carbon emissions (177.73 kg/m^3^), offering a high strength-to-impact efficiency. To validate its practical applicability, a full-scale RE wall was constructed using the CDW30–C2 mixture and subjected to thermal insulation tests in a controlled climate chamber at 40–80% relative humidity. The findings demonstrated a time lag of up to 90 min and a decrement factor of 0.85, indicating favorable thermal inertia and effective moderation of heat transfer. The synergistic effects of CDW particles enhanced mechanical interlocking and matrix densification, while CaO contributed to pozzolanic reactivity and void filling. Compared to conventional fired brick and concrete, the optimized RE mix demonstrated competitive performance with significantly lower environmental impact. These findings demonstrate the viability of CDW–CaO stabilized rammed earth as a climate-resilient, low-carbon, and resource-efficient building solution for sustainable construction.

## Introduction

Globally, the construction sector is a significant contributor to carbon emissions, accounting for approximately 38% of total CO_2_ emissions from the construction process across the supply chain^1,2^. Because of these significant environmental issues, there has been an increase in the demand for low-carbon construction materials consistent with sustainability and the principles of a circular economy. The construction material rammed earth (RE) is regaining popularity as a traditional method that is eco-friendlier responsive than the high-carbon construction materials^3–6^. The construction method involves compacting soil mixtures into construction formwork to create monolithic wall construction. The environmental benefits of RE construction are grounded in the use of locally-sourced construction materials with low/impact energy input, resulting in low environmental impact, and its great thermal mass allows for passive temperature regulation in buildings^3,7^. Through life cycle assessments (LCA), it has been determined that RE walls can lower embodied energy up to 70% and carbon emissions by over 90%, when compared to current concrete or masonry systems^1,4,8,9^.

Regardless of these benefits, the applications of RE in modern construction are limited by certain technical constraints, most importantly, its relatively low mechanical strength, especially compressive strength, which can be affected by moisture content, and compaction quality^3,4,7,10^. The most often utilized RE stabilizers in both laboratory and practical applications are lime^11–13^ and cement^14–16^. But because these binders are not sustainable, more environmentally friendly materials have been suggested as alternatives to cement and lime in stabilized RE structures. These include fly ash^3,14^, rice husk ash^17,18^, glass waste powder^19,20^, and other wastes^15–17,19^. Stabilizers can enhance the unconfined compressive strength (UCS), moisture resistance, and durability of RE walls^7–10,21^. However, their use contradicts the philosophy of low-carbon construction; cement makes up around 8% of global anthropogenic CO_2_ emissions, as cement is made in high-temperature kilns to produce clinker^8,22,23^. Therefore, due to these impacts, researchers are investigating new stabilizers made from industrial by-products and natural minerals with lower carbon footprints^9,10,22,24,25^. For example, Siddiqua et al.^26^ assessed the impact of calcium carbide residue and class-F fly ash on local fine soil in order to enhance it for RE construction. Calcium carbide residue is utilized as a soil binder due to its characteristics, which include its small particle size and high specific area. After 3 to 60 days of curing, the strength dramatically increased from 0.2 MPa to 5 MPa. Another study by Naeini et al.^27^ examined the effects of cement, calcium bentonite rammed earth, and wood fly ash. With a 28-day compressive strength of 3.56 MPa and exceptional freeze–thaw resistance (92% strength retention after 12 freeze–thaw cycles), they stated that the ideal composition consisted of 5% fly ash, 5% cement, and 15% bentonite. Construction and demolition waste (CDW), which represents a substantial fraction of municipal solid waste in various parts of the world, is a potentially suitable resource to fulfill part of the virgin soil component of RE^25,28,29^. CDW, which is largely made up of silicates, oxides, and carbonates, can add weight and pozzolanic activity to the RE material, adding additional mechanical strength and thermal performance^30,31^. Another resource of interest is calcium oxide (CaO), which can be acquired through limestone powder that is a very reactive and abundant material with low embodied carbon when compared to traditional cement^7^. If the material is added properly, CaO can produce hydraulic and pozzolanic reactions that strengthen the RE matrix, without the carbon-producing hydration process associated with cement binding^13,32^. Research has indicated that CaO soil provides greater UCS and lower permeability relative to soils bound using traditional means, making CaO a viable low-carbon binder^7,13^. Nevertheless, because of CaO’s reactive properties, the material must be added in a carefully optimized amount, as excessive CaO will cause samples to shrink excessively or crack.

Despite the potential of CDW and CaO emerging, comprehensive studies regarding thermal and mechanical evaluations of stabilized RE walls utilizing either of these materials remain limited. The majority of previous studies have studied one performance measure, for example, thermal conductivity or UCS^3,9,10,33,34^, resulting in a limited understanding of the relationship between material, building performance, and environmental change. Furthermore, standardized testing regimes for modified RE formulations have not been developed. Inconsistencies in testing, such as sample size, curing, compaction energy, and duration of test administration, make it difficult to compare and replicate studies^16,35^. Therefore, developing replicable protocols directed at RE construction is valuable for informing practice and policy. Consequently, this research intends to evaluate the mechanical, thermal and microstructural properties of RE composites with varying proportions of CDW (10–30% by weight) and CaO (2–6% by weight). This study assesses how the weight percentages of CDW and CaO influence compaction characteristics, UCS, and thermal performance through time lag, decrement factor (DF), and peak internal temperature (PIT) under constant humidity conditions. Microstructural and mineralogical changes were evaluated using scanning electron microscope (SEM) and X-ray diffraction (XRD) to explain the mechanisms of stabilization. This study will contribute to better understanding of how CDW can be sustainably reused in construction. The study will provide knowledge to identify acceptable mix proportions suitable for strength, durability, and thermal performance.

## Materials and methodology

### Materials

#### Soil

**Fig. 1:**
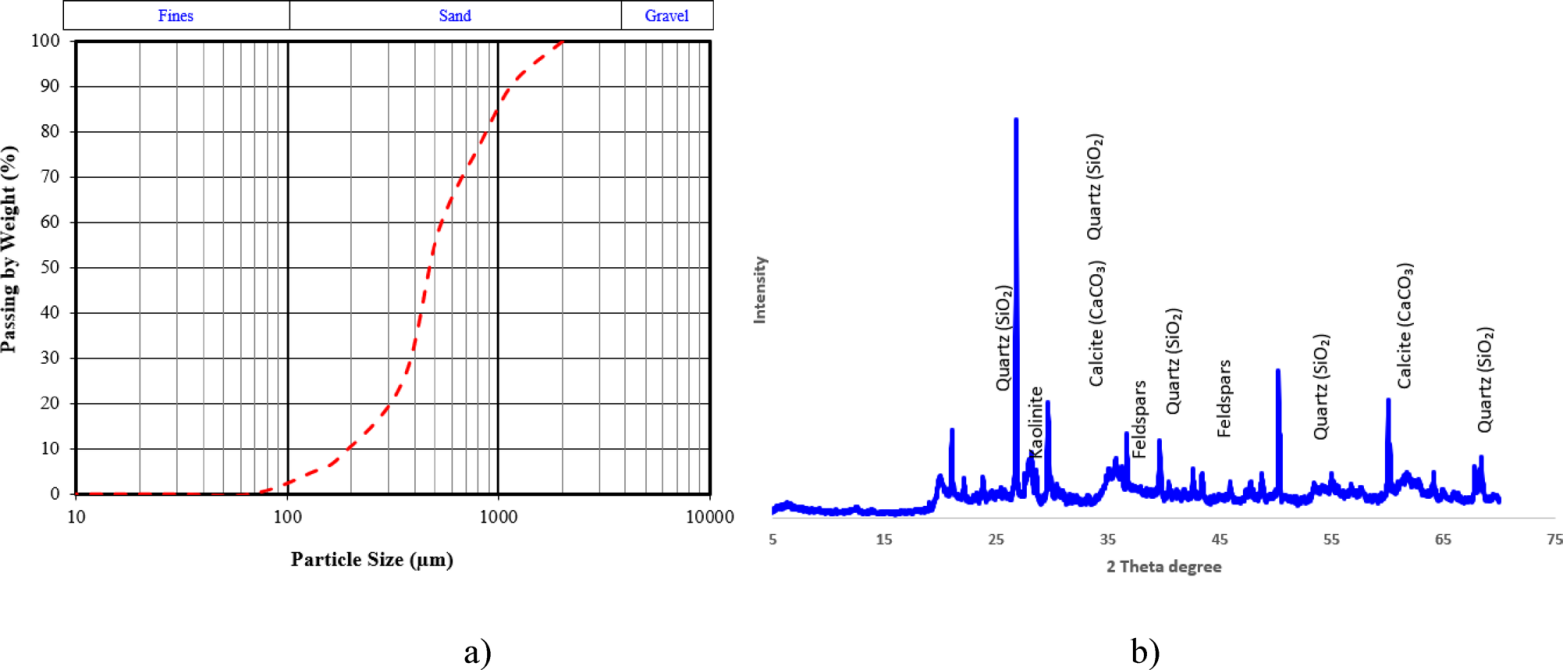
(**a**) Particle size distribution of the soil used and (**b**) XRD of soil used.

The soil used in this investigation (obtained from Mansoura city, Egypt) was a medium-grained natural inorganic sandy soil having the mechanical properties of rammed earth composites. The soil was dried in an oven at 60 °C until a constant mass was achieved and underwent sieve analysis to assess the particle-size distribution (PSD). The PSD curve plotted in Fig. 1a, shows that the soil has a sandy character with rather small amount of fines, < 1.5%, and a D50 of about 0.43 mm, and it was well classified according to the Unified Soil Classification System, ASTM D2487^36^, as a poorly graded sand. A dry sample completed for conducting the UCS tests was prepared by sieving out particles greater than 2 mm^4^. The X-ray diffraction (XRD) analysis of the soil showed that quartz (SiO_2_) is the dominating phase, which aligned heavily with the sandy character of the soil, shown in Fig. 1b. Minor peaks corresponding to feldspar minerals, such as albite and orthoclase, were also identified, indicating the presence of silicate-based components. Trace amounts of kaolinite and calcite were detected, suggesting minimal clay and carbonate content, which aligns with the particle size distribution results showing fines below 1.5%.

#### Cement

**Fig. 2:**
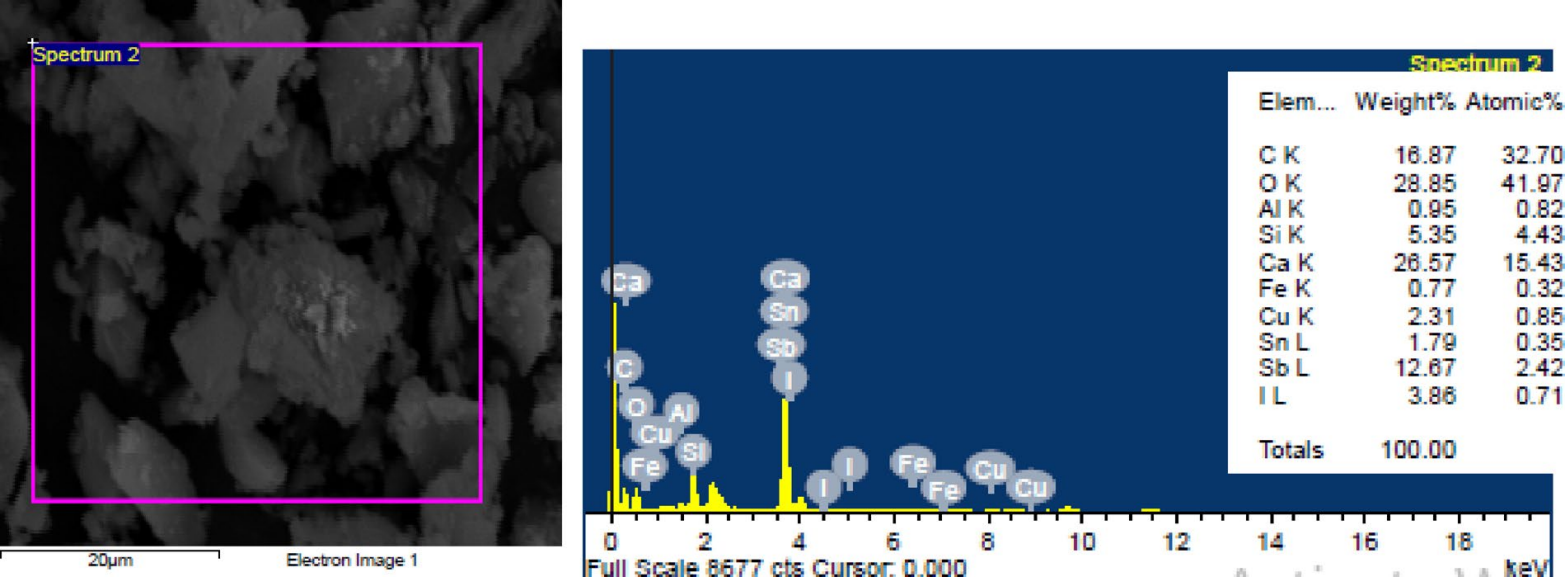
SEM and EDX results of Portland cement.

In this study, Portland cement (PC) CEM I 42.5 N was utilized in accordance with EN 197-1/2011. The performance of CDW-stabilized RE was evaluated against conventionally constructed RE, where Portland cement sourced from a local supplier served as the binder for cement-stabilized RE samples. Figure 2 presents the SEM image and energy-dispersive X-ray (EDX) spectroscopy of the cement, revealing that it is predominantly composed of irregular clinker particles with elevated levels of calcium (Ca) and silica (Si).

#### Construction and demolition waste

**Fig. 3:**
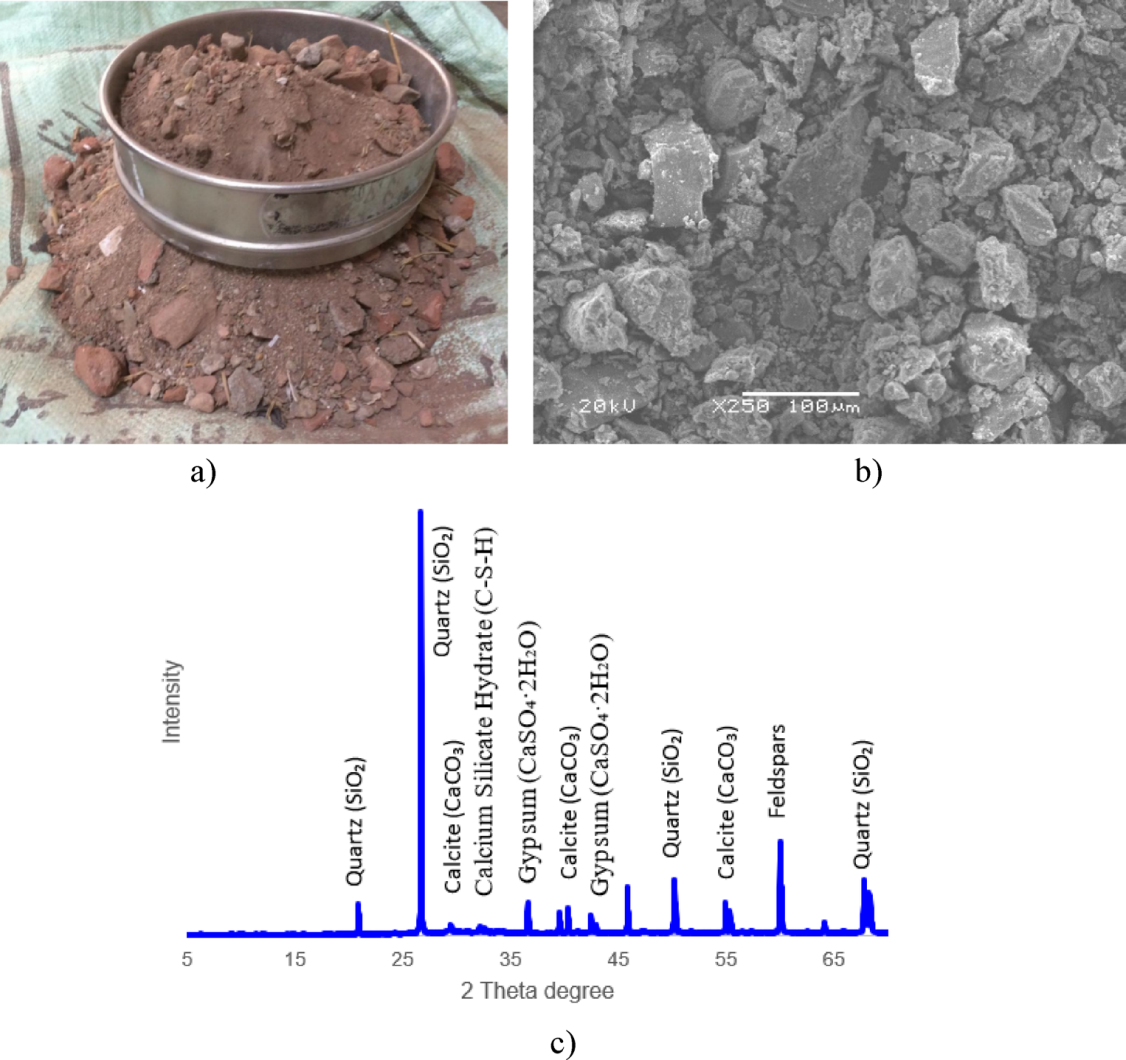
CDW: (**a**) Macro scale, (**b**) SEM image, and (**c**) XRD.

CDW, primarily in the form of recycled concrete aggregate (RCA), was sourced from demolished structures located across several demolition yards. The collection and processing of this material were conducted to produce a representative CDW material for experimental investigations, as presented in Fig. 3a. The RCA predominantly consists of sand and gravel-sized particles, with aggregate sizes ranging between 0.6 mm and 19 mm. Specimens were prepared using RCA with full particle gradation, and in combination with soil and stabilizers^28,29^. In mixed specimens, RCA particles larger than 19 mm and smaller than 0.6 mm were excluded to better control the final granulometric composition. The scanning electronic microscope (SEM) micrograph of the RCA, shown in Fig. 3b, reveals an irregular, angular morphology with rough surface textures and fractured edges and features typical of mechanically crushed concrete. These characteristics are beneficial for mechanical interlocking and potential chemical bonding with stabilizers. XRD analysis of the CDW, shown in Fig. 3c, confirms the mineralogical composition, indicating a dominant presence of Quartz (SiO_2_) and Calcite (CaCO_3_), along with notable peaks of Gypsum (CaSO_4_·2H_2_O) and Calcium Silicate Hydrate (C–S–H), the latter suggesting the partial hydration products of cement. Minor phases identified include Anorthite (CaAl_2_Si_2_O_8_), linked to ceramic or brick inclusions, and Larnite (Ca_2_SiO_4_), which reflects residual un-hydrated cementitious components^23,29^.

#### Calcium oxide (CaO)

**Fig. 4:**
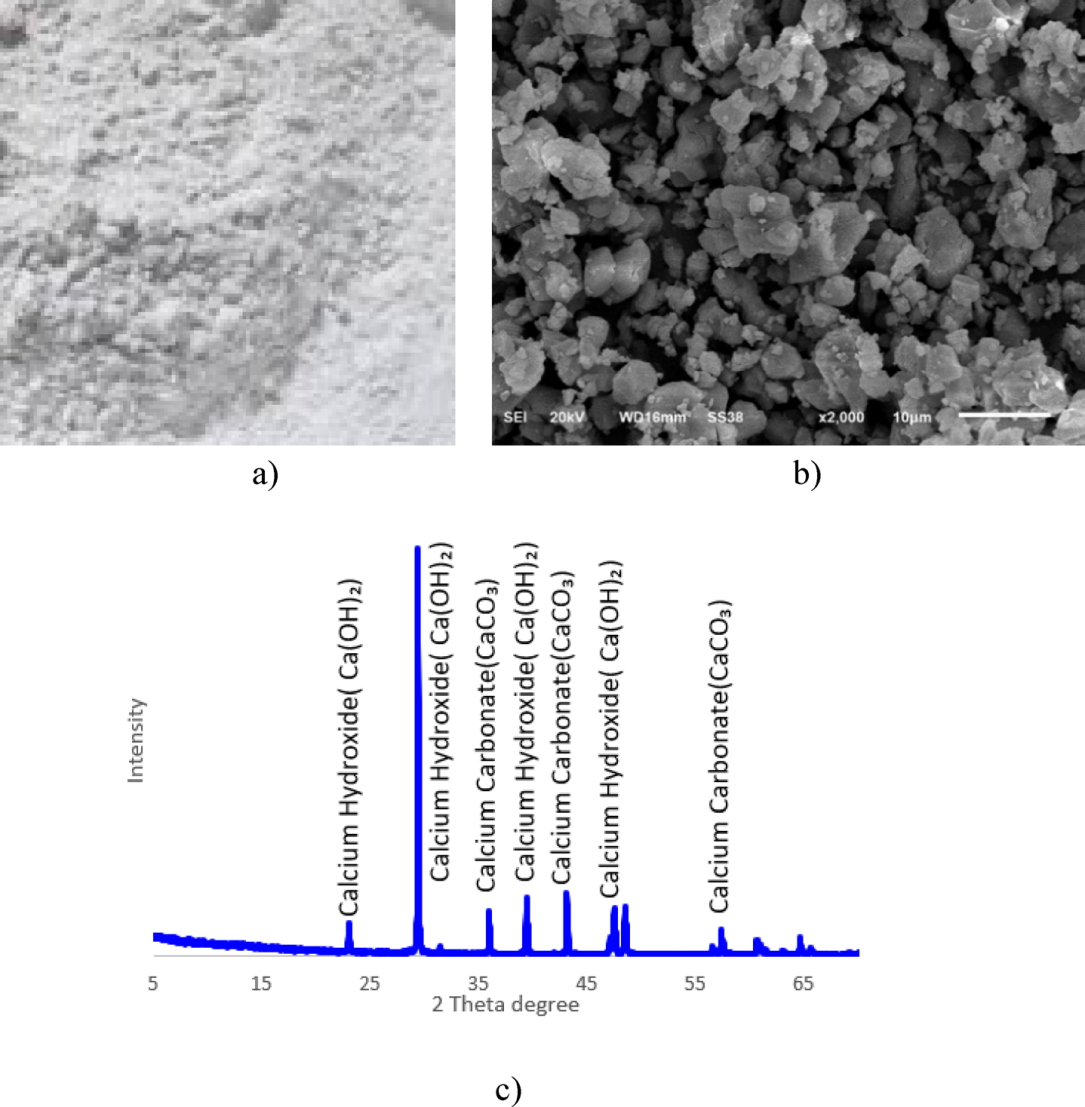
SEM and XRD results of calcium oxide.

The stabilizer utilized in this study is calcium oxide (CaO), derived from ultra-finely ground limestone deposits located in Helwan, Egypt. The raw material shown in Fig. 4a, commonly known as lime powder, primarily consists of calcium carbonate (CaCO_3_) and is processed through calcination to produce calcium oxide, the active stabilizer. Calcium oxide enhances the performance of CDW-stabilized RE systems by chemically reacting with water and soil constituents (e.g., clay or silica) to form binding compounds such as (C–S–H) or calcium aluminate hydrate (C–A–H), in addition to physically occupying inter-granular voids to improve soil compactness. The calcined calcium oxide exhibits a specific gravity of 2.7 and an un-compacted bulk density of 0.9 g/cm^3^, though these values are based on the processed material and may vary with calcination conditions. The SEM image of the powder is presented in Fig. 4b, while its chemical and mineralogical properties are detailed in Table 1, respectively. XRD analysis of the calcined product, as presented in Fig. 4c, confirms a dominant composition of calcium oxide (CaO), with residual minor phases potentially including quartz, albite, kaolinite, and microcline, depending on the extent of calcination and impurity retention from the original limestone (98.15% CaCO_3_ prior to calcination).

**Table 1:** Chemical and mineral composition of the CaO.

Chemical composition								
SiO_2_	Al_2_O_3_	CaO	Fe_2_O_3_	MgO	SO_3_	SrO	CO_2_	P_2_O_5_
0.0979%	0.188%	57.5%	0.0138%	0.115%	0.0121%	0.0083%	42.1	0.0122

Mineral composition							
Quartz	Calcite	Illite	Kaolinite	Dolomite	Chlorite	Quartz	Calcite
0.09%	98.15%	0.75%	0.94%	0.02%	0.05%	0.09%	98.15%

### Sample preparation and mixture design

The constituent materials, natural soil, CDW, and CaO were carefully prepared for testing. To ensure uniform conditions, all materials were oven-dried at 100°C for 24 h and subsequently stored in airtight containers to avoid unintended moisture absorption^14,17,26,27^. Based on the measured moisture content and known mold dimensions, precise quantities of soil, CDW, CaO, water, and a constant percentage (5%) of Portland cement were calculated for each mix. All dry materials were weighed using a digital balance with 0.01 g precision. Homogenization was achieved using a mechanical mixer, where the predetermined water was gradually added to ensure optimal workability and even moisture distribution. Sample consistency was addressed by utilizing a layer-by-layer compaction process that compacted each layer to a constant height to obtain the same density and structure throughout all samples^12,15,37^. This method minimizes variance with samples and obtains at least three replicates for each mixture. Before the samples preparation, the target values of MDD and OMC were established using the Proctor compaction test in accordance with BS 1377 (1990) Part 2. After the samples were compacted, they were allowed to set for 24 h before demolding. The samples were left to cure under reasonable conditions for stabilization. There were two curing timeframes: for samples cured for up to 7 days, samples were placed into a humid chamber (80% relative humidity and 22 °C) for 6 days, then taken out to cure at room temperature for 1 day; for samples cured for up to 28 days, samples were placed in a humid chamber at 80% relative humidity and 22 °C for 21 days, then taken out to cure at room temperature for 7 days. To ensure statistical validity and repeatability of results, three replicate specimens were prepared and tested for each mix design, as detailed in Table 2, which outlines the variables of the mixture design^22,23^.

**Table 2:** Variables of mixture design.

Mix ID	CDW (%wt)	CaO (%wt)	Description
REF	0	0	Reference mix
CDW10-C2	10	2	Low CDW, Low CaO
CDW10-C6	10	6	Low CDW, High CaO
CDW30-C2	30	2	High CDW, Low CaO
CDW30-C6	30	6	High CDW, High CaO
CDW30-C4	30	4	High CDW, Medium CaO
CDW20-C6	20	6	Medium CDW, High CaO

### Sample preparation for thermal properties

To evaluate the peak temperature, decrement factor, and time lag associated with each mix composition, a custom mold was designed and fabricated to ensure precision and uniformity in sample production^23^. The thermal performance specimens were compacted using a hydraulic press to achieve consistent density across all layers. Each sample was shaped into a cube with dimensions of 150 mm × 150 mm × 100 mm, with compaction performed in ten uniform layers. Each layer was compacted at its designated OWC and MDD values for the corresponding mix designs. After compaction, the samples were allowed 48 h of curing in the moulds to stabilize their structure, after which a controlled curing process was applied to simulate real-world stabilized earth: first the primary curing phase was to place the samples in humified chamber for 21 days at 22 °C and 80% relative humidity, the next phase was a secondary conditioning phase for an additional curing period of 7 days under ambient laboratory conditions, to finish thermal preparation. To ensure statistical accuracy and repeatability of the thermal performance data, three identical samples were prepared and tested for each of the mixtures studied.

### Experimental testing

#### Unconfined compressive strength (UCS) test

ASTM D4219-02^38^ was followed in the UCS testing on cylindrical samples that were 40 mm in diameter and 80 mm in height, with a diameter/height ratio of ½. The UCS tests were conducted at a constant vertical strain rate of 1% per minute using a GDSLF10 load frame with a 10 kN capacity. UCS test was performed after 7 and 28 days of curing to examine the impact of curing time on mechanical strength.

#### Thermal performance evaluation

**Fig. 5:**
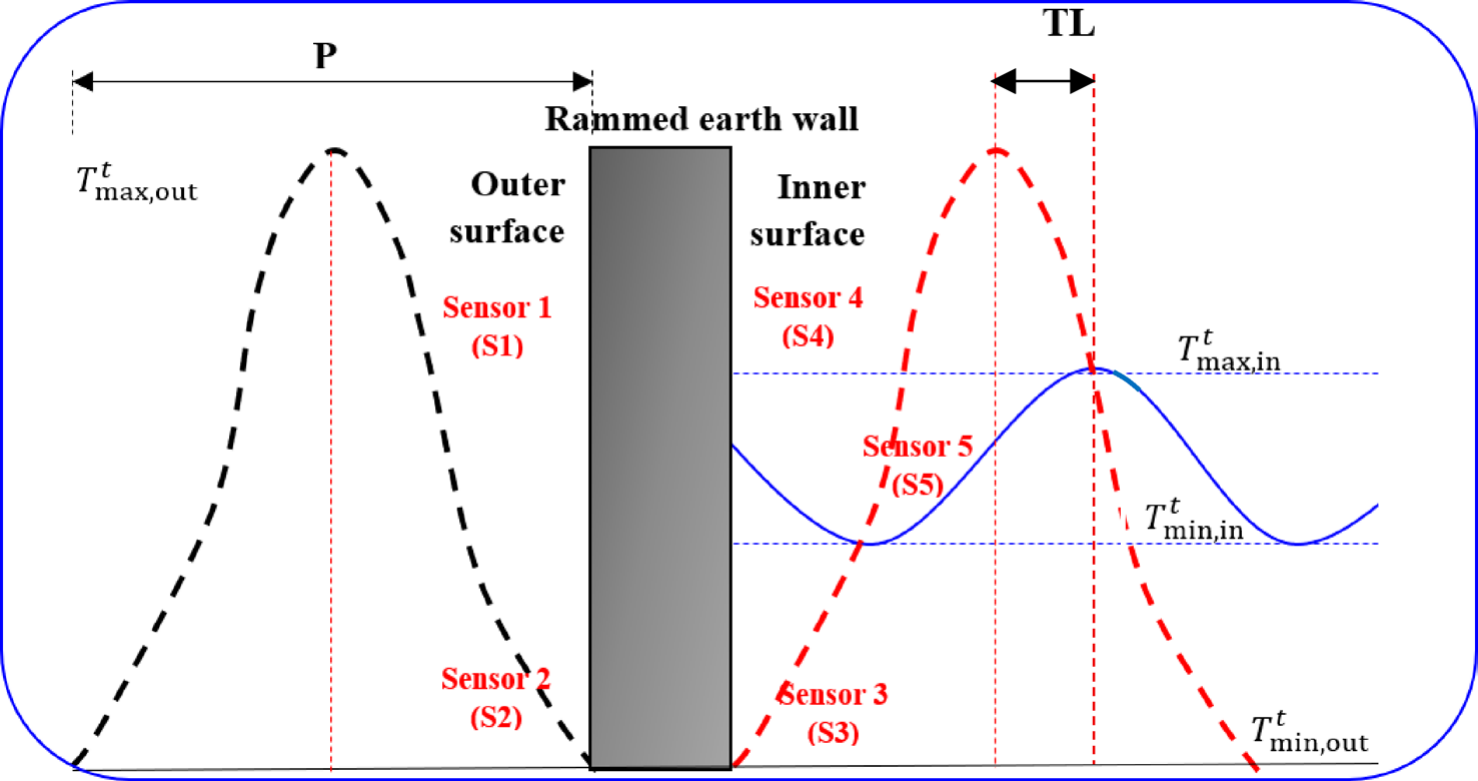
The schematic view of time lag and decrement factor.

Time lag (TL) and decrement factor (DF) have been a focus of thermal performance evaluation of building envelopes in recent years. The peak internal temperature (PIT) is the highest temperature that occurs at the wall inner surface during the heating cycle and reflects the ability of the wall to resist heat transfer to maintain cooler interior conditions. TL is illustrated in Fig. 5 and is defined as the time it takes for the peak of the temperature wave to propagate from the outer surface of a wall to the inner wall surface of the wall. The DF is defined as the ratio of the temperature fluctuation amplitude at the inner surface of the wall to the temperature fluctuation amplitude of the outer surface of the wall. Therefore, the thermal performance of the stabilized RE walls can be evaluated by measuring three parameters: TL, DF, and peak internal temperature (PIT), which provide insight into the insulation and thermal inertia of the wall systems.

**Fig. 6:**
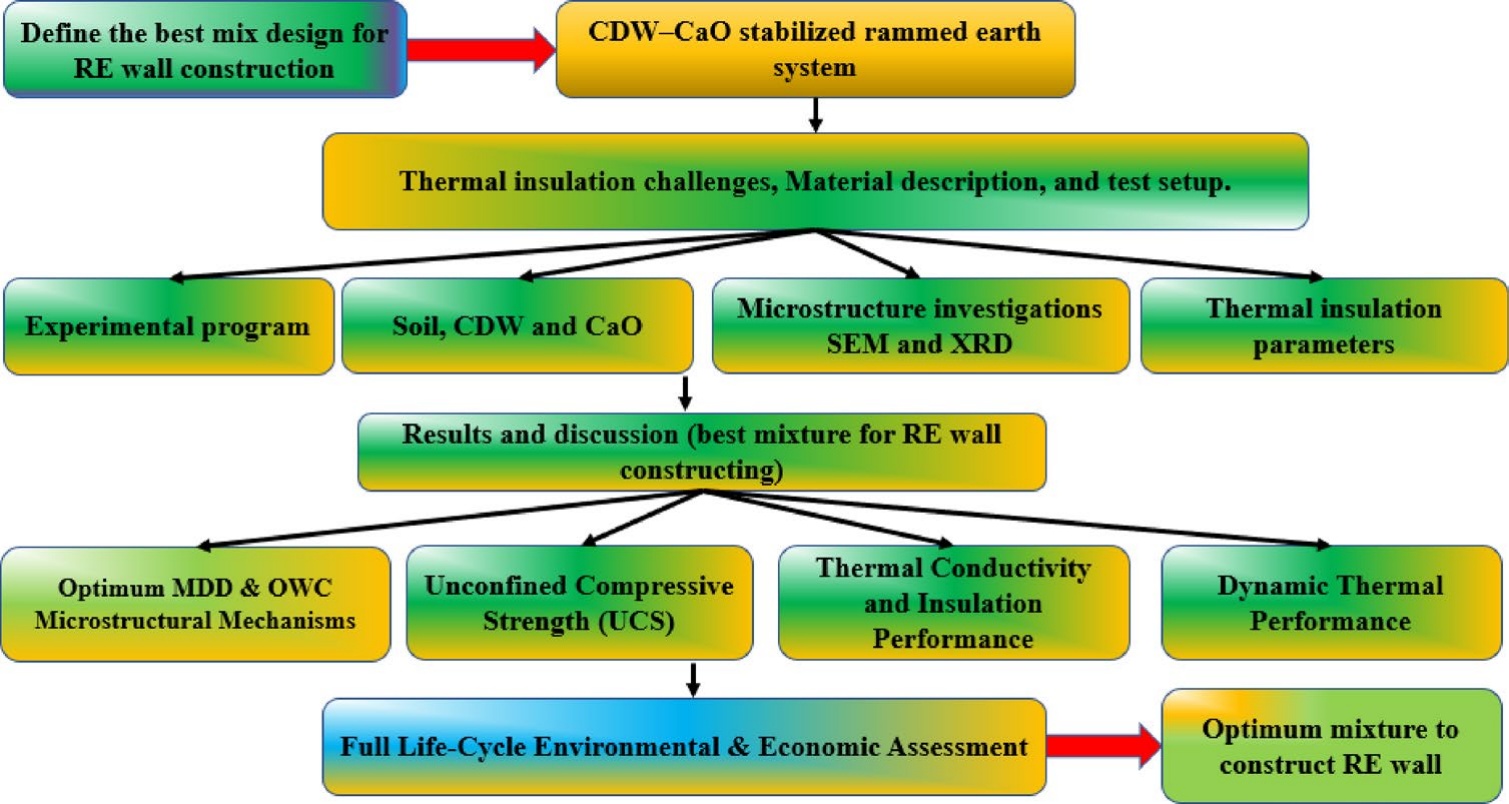
Schematic overview of CDW–CaO stabilized rammed earth system.

As shown in Fig. 6, the TL is the delay between the external temperature peak and the internal temperature peak and it was calculated using the following Eq. (1):

1 $${\text{TL}} = T_{{{\text{(max,}}\,{\text{in}})}}^{t} - T_{{(\max ,\,{\text{out}})}}^{t}$$

where; T^t^_max, in_ and T^t^_max, out_ represent the times to reach the maximum internal and external temperatures, respectively. The DF reflects the dampening of thermal amplitude through the wall and is given by Eq. (2):

2 $${\text{DF}} = \frac{{T_{{{\text{max}},\,{\text{in}}}} - T_{{{\text{min}},\,{\text{in}}}} }}{{T_{{{\text{max}},\,{\text{out}}}} - T_{{{\text{min}},\,{\text{out}}}} }}$$

where; ${T}_{\text{max},\text{ in}} and$ ${T}_{\text{min},\text{ in}}$ are the maximum and minimum internal temperatures, respectively; and ${T}_{\text{max},\text{ out}}, and$ ${T}_{\text{min},\text{out}}$ are the maximum and minimum external temperatures, respectively. A higher TL is desirable as it indicates improved thermal insulation and heat retention, while a lower DF signifies superior attenuation of temperature fluctuations, ensuring greater internal thermal stability. Figure 6. summarizes the experimental workflow, from defining the optimal CDW-CaO stabilized rammed earth mix to final RE wall construction.

#### Thermal testing setup

Thermal conductivity tests were conducted in a controlled environment climate chamber, designed to maintain precise temperature and humidity conditions with a tolerance of ± 0.3 °C. The tests were carried out under three relative humidity (RH) scenarios: 40% RH to represent dry conditions, 60% RH to represent semi-dry and semi-humid conditions, and 80% RH to represent humid conditions. Before testing, specimens were placed in a custom-built conditioning chamber for 24 h to stabilize their internal moisture content. During the testing phase, five digital temperature sensors, each with an accuracy of ± 0.5 °C, were installed: two on the outer surface, two inside the hollow core, and one at the midpoint depth of the wall section to capture thermal gradients more accurately. Temperature data was recorded continuously, providing real-time thermal profiles throughout the heating cycle.

#### Microstructure investigations

Through detailed examination using SEM analysis and EDX spectroscopy, microstructural understanding was obtained for the various RE design mixtures analyzed to discover how the underlying stabilization mechanisms operated. The SEM imaging was achieved from a high-resolution, field-emission SEM instrument under a voltage of 15–20 kV to give good detail of surface morphology, as well as particle bonding features. Furthermore, XRD was completed to assess the mineralogical and crystalline phases in the RE samples prior to and following treatment. XRD patterns were collected using PANalytical X’pert PRO, from Cu-Ka radiation (λ = 1.5406 Å) over a range of 2θ from 5° to 70°, at a scanning speed of 2°/min.

## Results and discussion

### Compaction test

**Fig. 7:**
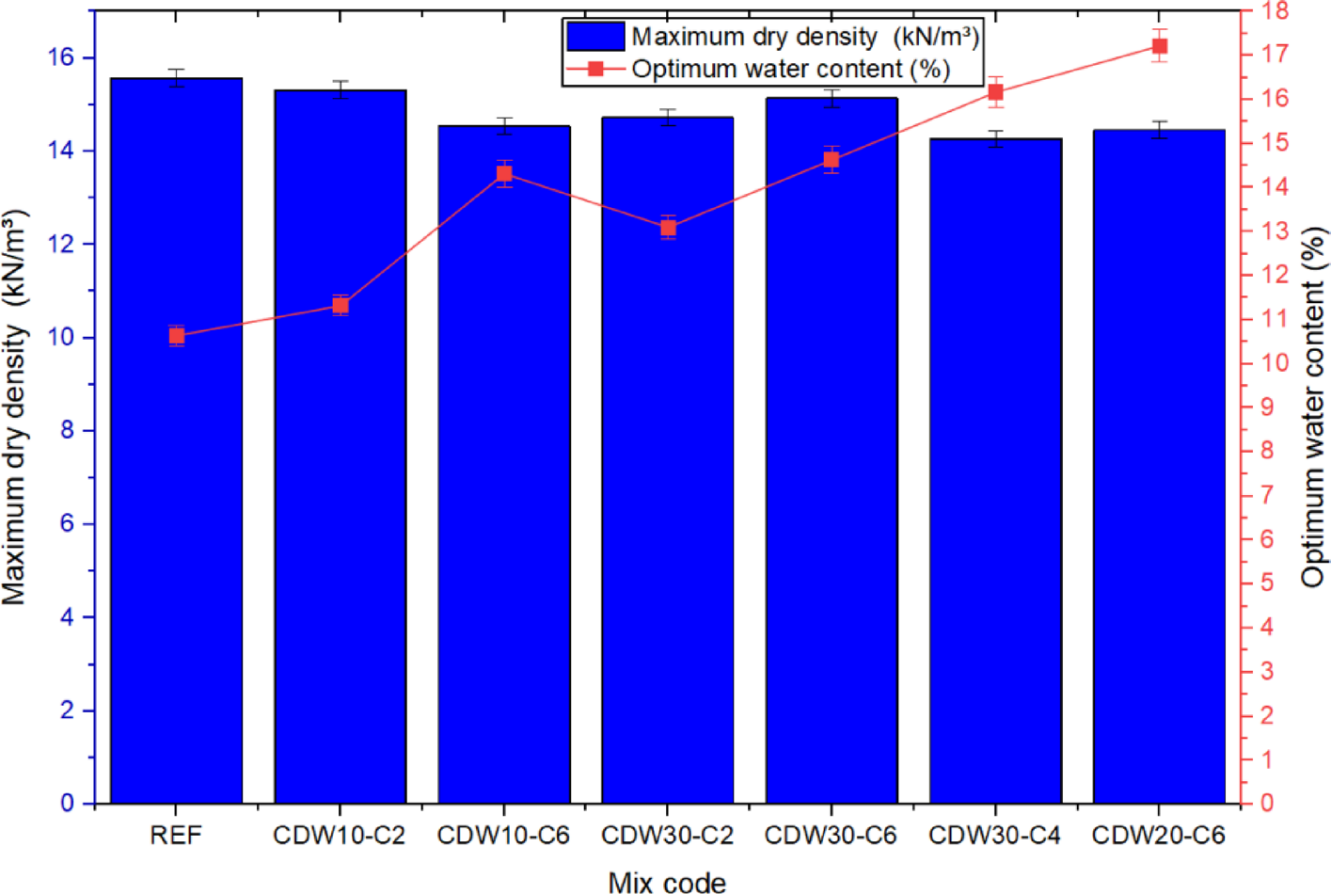
Results of OWC and MDD for different CDW–CaO mix designs.

The compaction behavior of the rammed earth mixtures, influenced by the incorporation of CDW and CaO, revealed a complex interplay between particle morphology, water demand, and chemical reactivity, as presented in Fig. 7. Compared to the reference mix composed of natural sandy soil, which recorded the highest MDD of 15.57 kN/m^3^ and a relatively low OWC of 10.64%, the inclusion of CDW generally resulted in a reduction in MDD and a corresponding increase in OWC. For example, the CDW10-C2 mix, with 10% CDW and 2% CaO, showed a slight drop in MDD to 15.31 kN/m^3^ and an increase in OWC to 11.32%, highlighting the water-absorptive nature of recycled concrete aggregates^12,19,27,32^. Increasing the CaO content to 6% in the CDW10-C6 mix further reduced MDD to 14.54 kN/m^3^ and increased OWC significantly to 14.32%, reflecting the role of fine, reactive lime particles in elevating water demand. Notably, the CDW30-C6 mix (30% CDW and 6% CaO) exhibited a moderate MDD of 15.14 kN/m^3^—almost comparable to the reference—suggesting that at higher stabilizer content, improved binding and void filling can partially offset the density loss from angular CDW particles. Conversely, CDW30-C4 and CDW20-C6 showed the lowest MDDs (14.27 and 14.45 kN/m^3^, respectively) and the highest OWCs (16.17% and 17.22%), emphasizing that excessive CaO content can lead to inefficient compaction due to excess water demand. The results differ from both previous research into how cement- and fly ash-stabilized soils behave, typically higher cement part content increases MDD and reduce the OWC due to improved aggregative^11,16,37^, while in regard to fly ash it behaves opposite of cement increasing OWC and decreasing MDD due to the generally finer texture and lower specific gravity^3,14,28^. The responses seen in the CDW–CaO systems reported here represent a more complicated response, establishing various interactions between aggregate interlock, reactivity, and packing density, which were in alignment with previous research^9^ in which the spatial orientation governed particle geometry, additive properties, and that they are vital to evaluating compaction of earth materials.

### Unconfined compressive strength of rammed earth

**Fig. 8:**
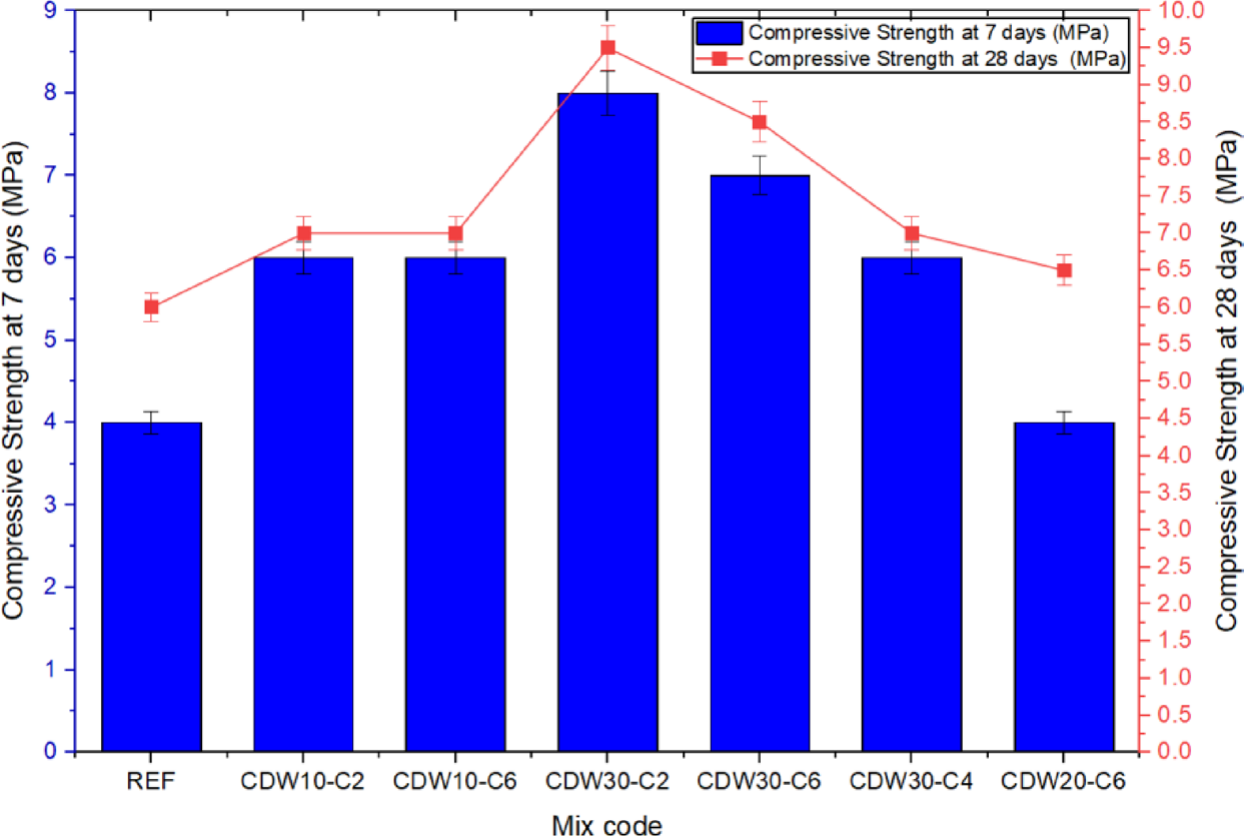
UCS of RE samples at 7 and 28 days for different CDW–CaO mix designs.

The UCS results shown in Fig. 8 increased considerably with the use of CDW and CaO. Mix CDW30-C2 mix showed the highest strengths after 7 (8 MPa) and 28 (9.3 MPa) days of curing compared with the REF mix (pure natural sandy soil) that showed the lowest strength (4 MPa), demonstrating that CDW and CaO stabilization is beneficial. This improvement likely stems from the physical interlocking provided by the angular CDW particles and the pozzolanic reactions triggered by CaO, which promote the formation of binding compounds. These findings align with studies by Bui et al.^8^ and Liu et al.^9^, who observed that recycled concrete aggregates enhance strength through increased internal friction and densification. However, increasing CaO from 2 to 6% in mixes with lower CDW content (e.g., CDW10-C6) yielded only a slight strength gain, suggesting that the reactive silica in the soil-CDW matrix may be insufficient to fully engage the higher CaO levels. This observation is consistent with findings reported by Martín-del-Rio et al.^11^, who noted that exceeding optimal lime content relative to available reactive materials can lead to diminished strength gains. The observed strength reductions in CDW20-C6 and CDW30-C4 emphasize the need for a balanced CDW and CaO proportion, as excessive CaO or CDW can impair bond formation and create weak interfacial zones due to over-saturation or inadequate reactive sites.

### Thermal conductivity

**Fig. 9:**
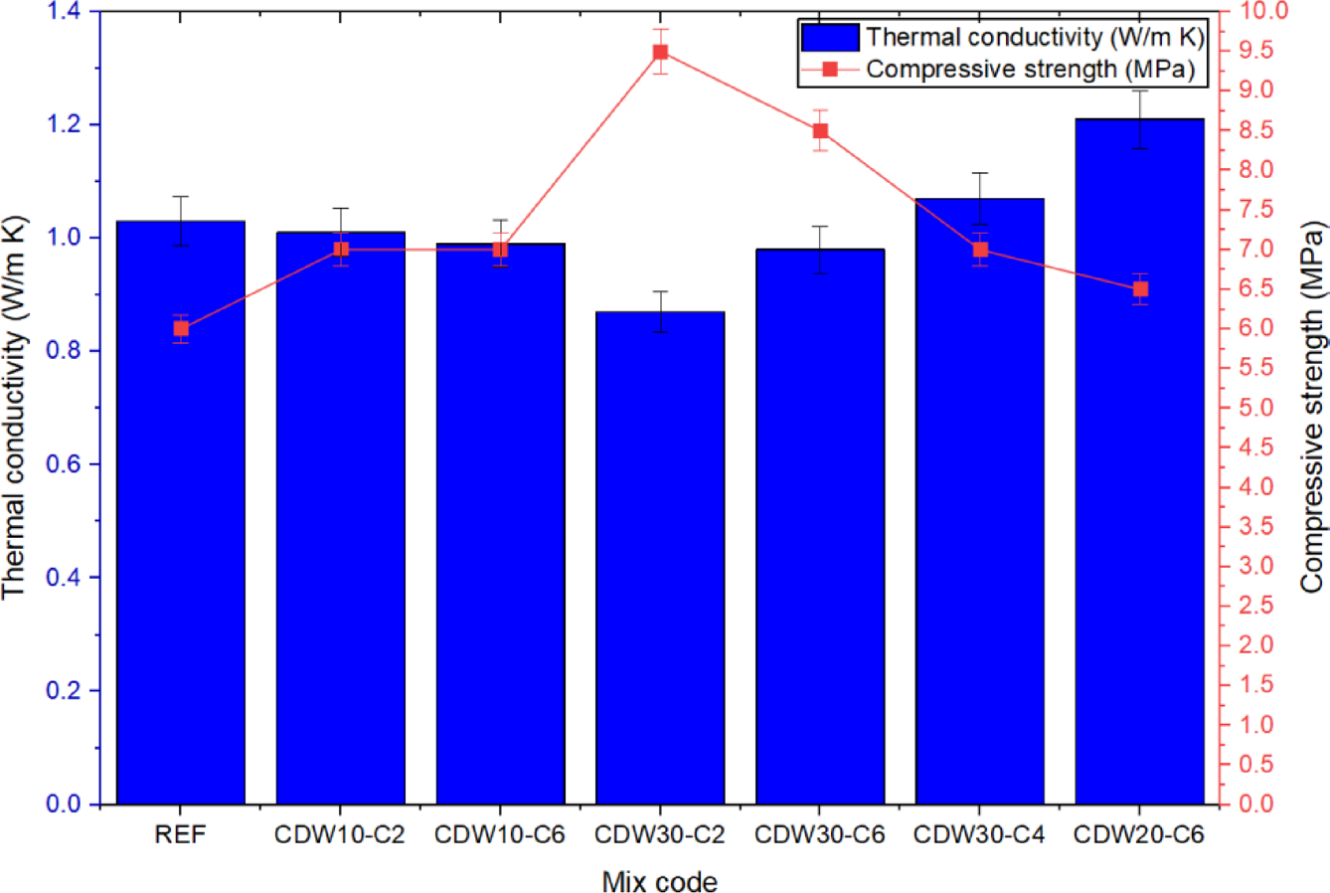
Comparison of thermal conductivity and UCS across various stabilized RE mixtures.

Figure 9 reveals an inverse relationship between UCS and thermal conductivity across most mixes, with the CDW30-C2 mix exhibiting the highest compressive strength (9.3 MPa at 28 days) alongside the lowest thermal conductivity (0.88 W/m·K), suggesting a denser microstructure enhanced by mechanical interlocking and reduced pore connectivity. Conversely, the CDW20-C6 mix, despite a moderate compressive strength (~ 6.5 MPa), displayed the highest thermal conductivity (1.22 W/m·K), likely due to the higher CaO content promoting the formation of thermally conductive phases like calcium hydroxide and C–S–H gels. This aligns with Ciancio et al.^12^ and Canivell et al.^7^, who noted that reactive lime can produce dense mineral phases that increase thermal conductivity, potentially compromising insulation properties. The REF mix, consisting of natural sandy soil, showed lower strength (4 MPa) and moderate thermal conductivity (1.03 W/m·K), indicating a lack of binding phases and thermal mass, consistent with Ávila et al.^10^, who emphasized the influence of density, moisture content, and mineral composition on thermal conductivity in earth materials. Compared to previous studies, Bui et al.^4^ reported thermal conductivities of 0.9–1.1 W/m·K for unstabilized rammed earth, similar to the REF mix, while stabilized mixes with cement achieved strengths up to 5 MPa with conductivities around 1.0 W/m·K. The current study’s CDW30-C2 mix surpasses these values, suggesting that CDW and CaO offer superior strength and thermal insulation. However, the elevated conductivity in CDW20-C6 contrasts with findings by Wang et al.^13^ and Ciancio et al.^12^, who observed stable thermal performance with higher lime content, highlighting the need for balanced stabilization to optimize both strength and insulation. These results demonstrate that optimal CDW and CaO combinations enhance mechanical performance and thermal inertia, supporting sustainable building applications.

### Microstructural and chemical characterization

**Fig. 10:**
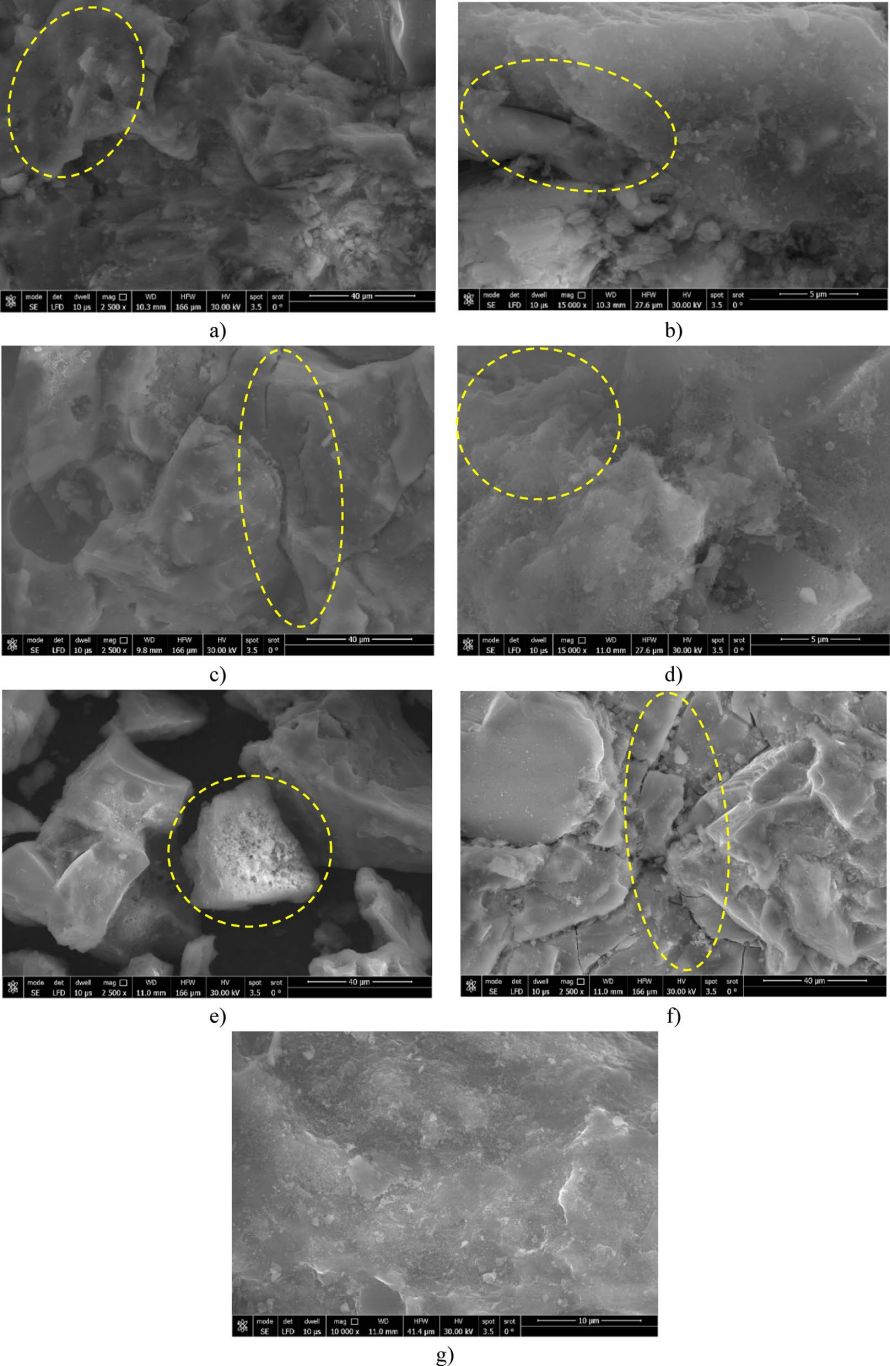
Microstructure of stabilized RE mixtures (**a**) REF, (**b**) CDW10-C2, (**c**) CDW10-C6, (**d**) CDW30-C2, (**e**) CDW30-C6, (**f**) CDW30-C4, (**g**) CDW20-C6.

A total of six RE mixtures, as well as the control mixture (REF), were analyzed using SEM imaging to assess the effect of CDW and CaO on microstructural development, see Fig. 10. The control mixture, consisting only of natural sandy soil, displayed a loose and porous matrix with ill-defined quartz grains and insignificant binder bridges. Such a morphology corresponds with its relatively low UCS of roughly 4 MPa and moderate thermal conductivity (1.03 W/m·K), suggesting poor particle cohesion and weak thermal dampening. Conversely, the CDW10-C2 mix (10% CDW, 2% CaO) evidenced distinct inter-particle interlocking mediated by angular CDW lithic fragments and early indications of cementitious phase formation, suggesting the beginning of CaO reactivity. This provided a marginal improvement in UCS and thermal resistance^11,17,39^. Increasing the amount of CaO from 2 to 6% in CDW10-C6 produced a noticeably denser microstructure. The increased amount of CaO caused better binding of CDW and filled the available voids with calcium-rich hydration products^11^. Therefore, while there still some relatively large localized voids, this indicates a surplus of CaO. This is consistent with the larger OWC and slightly lower MDD of 14.54 kN/m^3^, likely due to poor packing of the mix and variations in distribution of hydration. Conversely, CDW30-C2 had the densest and cohesive matrix from the other mixes, as this mix possessed adequate binding, mechanical interlocking, and an evenly distributed binder phase. These factors yielded the maximum UCS of 9.3 MPa seen in the results, and the lowest thermal conductivity (0.88 W/m·K) of the mixes seen in the results indicates more disconnection of pores in the microstructure, better thermal insulation capabilities. This mix showed adequate angularity of CDW and moderate reactivity of CaO.

When CaO was increased to 6% in CDW30-C6, it resulted in a structure that included high proportions of calcium-based compounds, which filled most of the inter-granular voids. However, the binder used led to large, trapped pores, which reduced the overall packing efficiency of the microstructure. This can be seen by the highest OWC of 16.17%, resulting in a moderate MDD of 15.14 kN/m^3^ and high thermal conductivity of approximately 1.22 W/m·K. It is suggested that high levels of CaO adversely affect thermal performance as it indicates a degree of microstructural coarsening. Alternatively, in CDW30-C 4, by using a 4% CaO addition, it appears that the level of cement could provide a balance between bond and density.

**Fig. 11:**
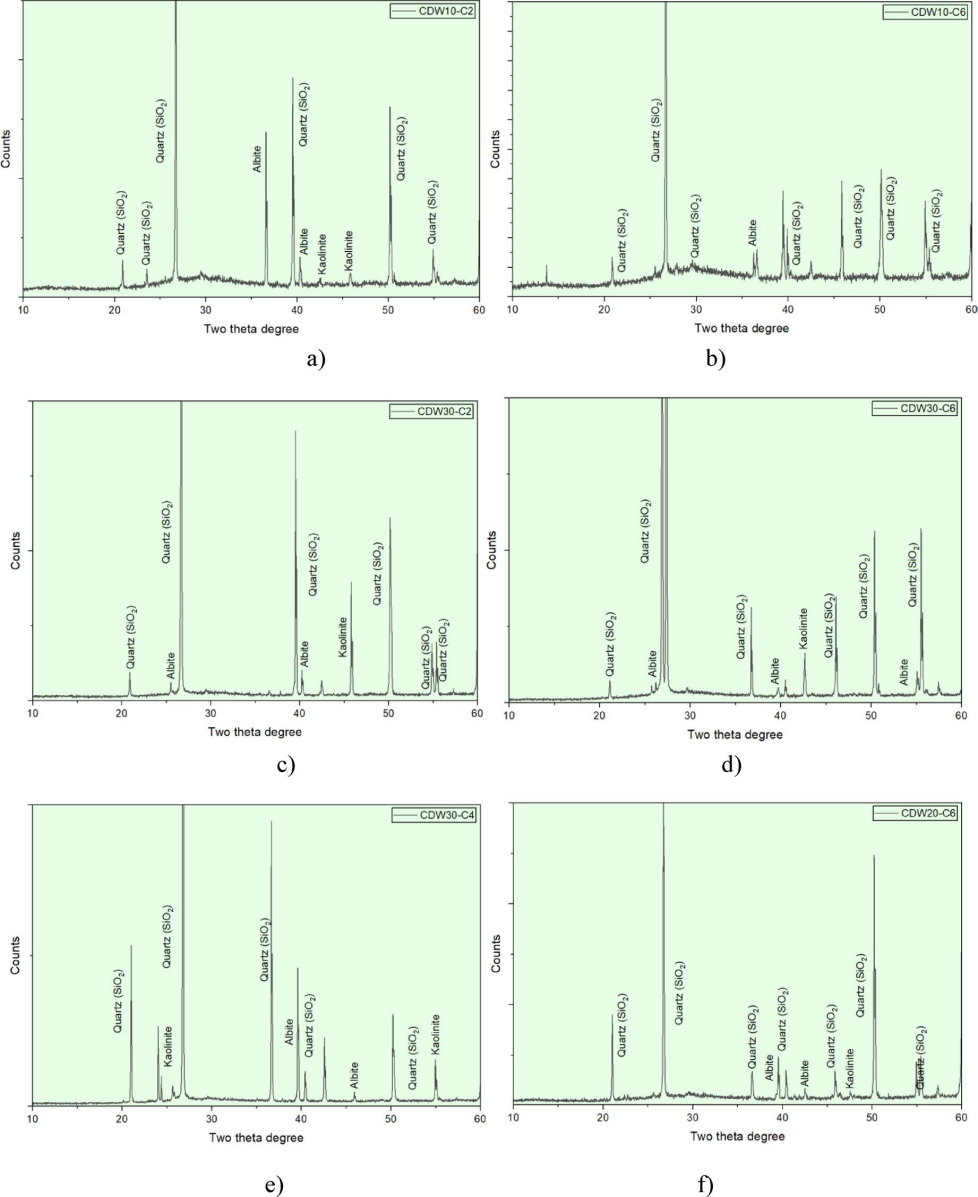
XRD of stabilized RE mixtures (**a**) CDW10-C2, (**b**) CDW10-C6, (**c**) CDW30-C2, (**d**) CDW30-C6, (**e**) CDW30-C4, (**f**) CDW20-C6.

XRD testing was carried out to study the changes in minerals with different amounts of CDW and CaO, as plotted in Fig. 11. In the CDW formulations containing CDW10-C2 and CDW30-C2, SiO_2_ clearly shows the sandy nature of the native soil through XRD. The SiO_2_ peaks measured at each phase appeared distinctly at 2θ = 20.9°, 26.6°, 36.6°, and 50.1°. The intensity of these peaks decreased as the CDW proportions increased. This reduction resulted from a dilution of the native sandy soil by the more amorphous CDW material, which makes up most of the CDW content^16,33^. Peaks related to feldspar minerals, specifically albite and orthoclase, were seen at 2θ = 21°, 27°, and 40–42°. These peaks match well with the alumino-silicate structure of the CDW material. This content improves the pozzolan’s effectiveness when activated with CaO. The second mineral phase formed was calcite (CaCO_3_), with peaks at 2θ = 29.4°, 39.4°, and 47.5°. This indicates that carbonation of the hydrated CaO content of the binder occurs, especially in the higher CDW mixes compared to the control mix. Kaolinite peaks at 2θ = 12.4° and 24.8° were of minor content, making up less than 1.5% of its clay fraction, which aligned with the clay fractions from PSD. In formulations with more CaO, such as CDW10-C6 and CDW30-C6, the peaks were much clearer at 2θ = 18°, 34°, and 47°. This suggests lime hydration and the formation of portlandite (Ca(OH)_2_). This mineral transformation relates to the beneficial formation of C–S–H and other binding phases, which enhance mechanical strength. It is important to note the potential significance of portlandite, as the larger amounts may explain the significantly higher OWC and the voids observed in SEM.

### Economic and environmental assessments

**Fig. 12:**
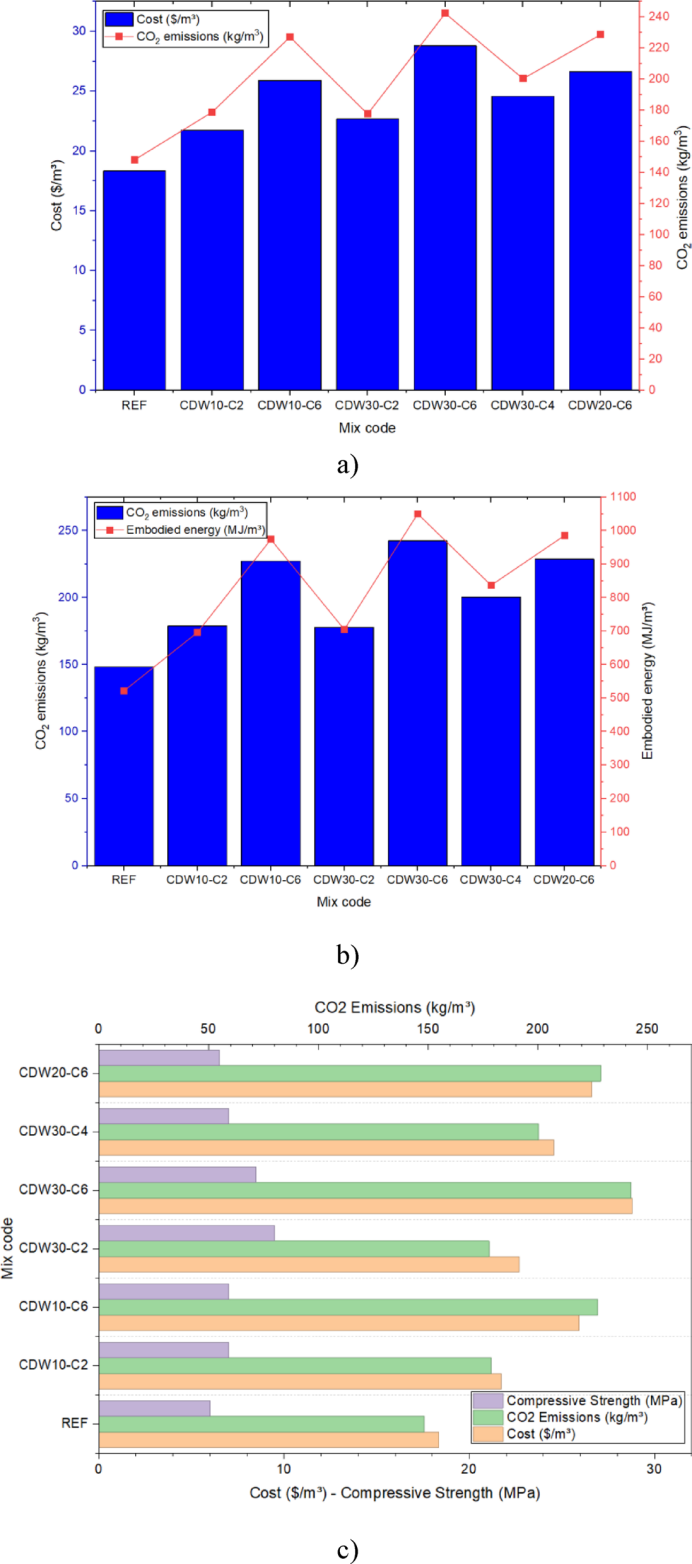
(**a**) Cost and CO_2_ emissions per 1 m^3^ for each RE mix design, (**b**) Embodied energy and CO_2_ emissions per 1m^3^ for each RE mix design, and (**c**) Cost, compressive strength and CO_2_ emissions per 1m^3^ for each RE mix design.

The economic and environmental assessments presented in Fig. 12 highlight the advantages and disadvantages associated with material substitution in rammed earth mixes, offering a cradle-to-gate life cycle assessment perspective. The control mix, composed solely of natural sandy soil, recorded the lowest cost ($18.8/m^3^) and CO_2_ emissions (148.23 kg/m^3^) as shown in Fig. 12a. Its mechanical performance remained the weakest (4 MPa), reflecting a limited capacity for structural enhancement without stabilization. The incorporation of CDW and CaO significantly increased both cost and emissions, with the CDW30-C6 mix exhibiting the highest CO_2_ emissions (240 kg/m^3^) and cost ($29.5/m^3^), driven by the energy-intensive processing of recycled aggregates and high cementitious stabilizer content, as detailed in Fig. 12a. This trend aligns with Min et al.^1^ and Huang et al.^2^, who observed that escalating binder content elevates both cost and emissions while improving durability. In contrast, the CDW30-C2 mix achieved the highest compressive strength (9.3 MPa) with moderate emissions (177.73 kg/m^3^), suggesting an optimal balance that minimizes environmental impact while maximizing performance, a finding corroborated by Fig. 12b and c. This mix supports the findings by previous research^22,23,28^, who demonstrated that strategic reuse of CDW can yield sustainable materials with a competitive environmental footprint.

The embodied energy assessment in Fig. 12b further reveals that mixes like CDW30-C6 approach 1000 MJ/m^3^, compared to the REF mix’s 500 MJ/m^3^, highlighting the energy demands of stabilizer processing. This is consistent with previous studies^39,40^, who advocated for integrating CDW with low-carbon binders like CaO to reduce environmental burdens while enhancing structural properties. Figure 12c illustrates that higher CaO content in mixes such as CDW20-C6 and CDW30-C6 sustains strong compressive strength but at a steeper environmental and economic cost, emphasizing the need for balanced material ratios. When compared to conventional materials, pure fired brick typically incurs CO_2_ emissions of 200–300 kg/m^3^ and costs of $30–$40/m^3^, with embodied energy ranging from 1000 to 2000 MJ/m^3^ due to high-temperature firing^31^. Concrete, on the other hand, exhibits emissions of 150–250 kg/m^3^, costs of $50–$70/m^3^, and embodied energy of 800–1500 MJ/m^3^, driven by cement production^41^. The CDW30-C2 mix, with its lower emissions (177.73 kg/m^3^) and cost ($22.68/m^3^) alongside comparable strength to brick, offers a more sustainable alternative. Figure 12c reinforces this by showing a favorable strength-to-emission ratio for CDW30-C2, demonstrating its potential for life cycle cost savings and reduced carbon footprint over the full building life cycle, from raw material extraction to construction.

### Thermal performance of CDW30-C2 RE-wall

**Fig. 13:**
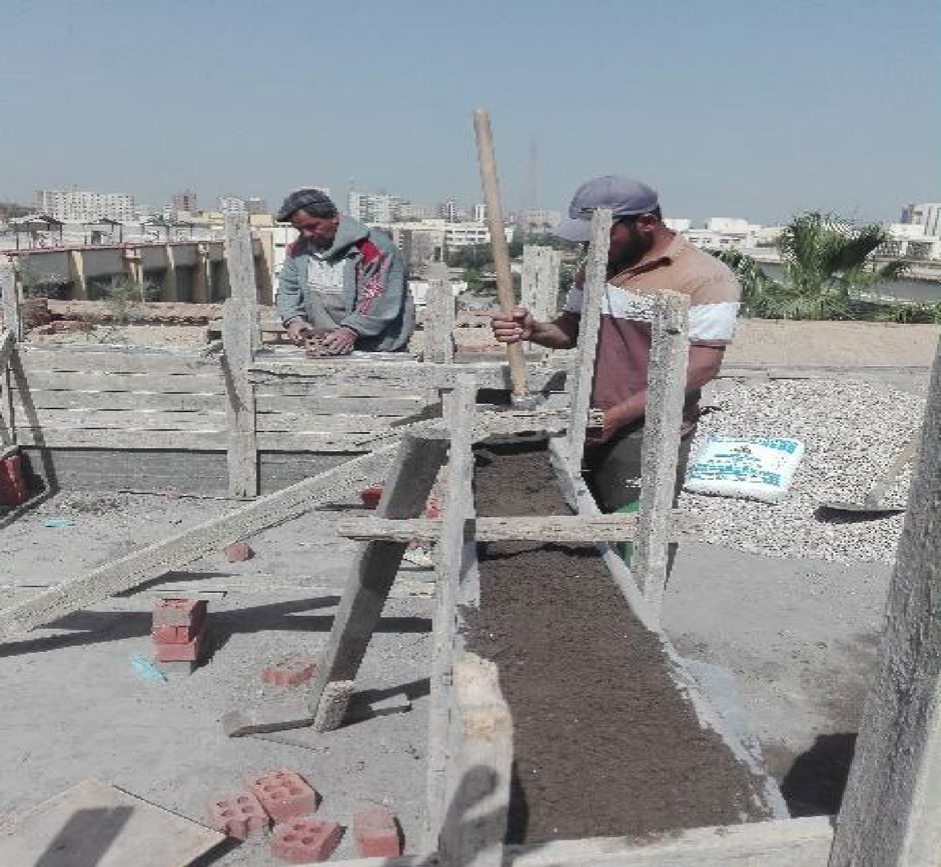
Preparation of the CDW30-C2 RE-Wall.

**Fig. 14:**
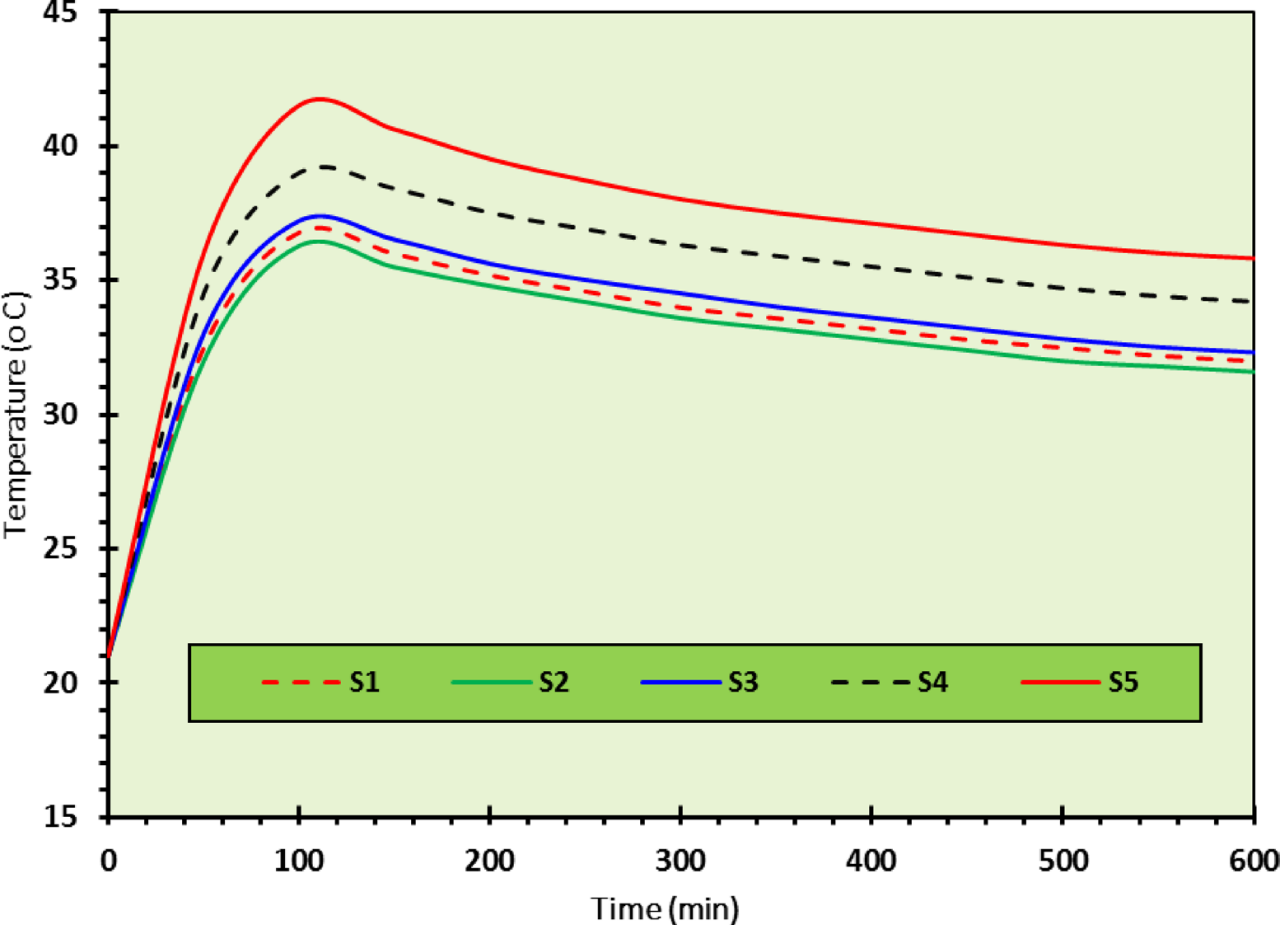
Thermal insulation test data for a hot climate with ideal humidity (temperature from 41 to 27 °C and 40% humidity).

**Fig. 15:**
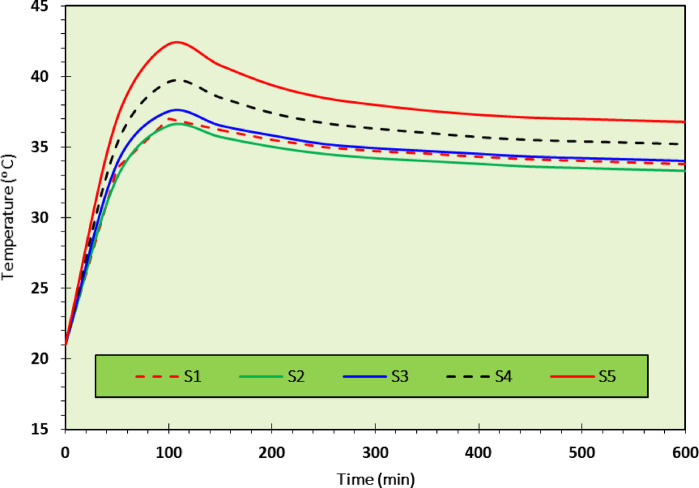
Thermal insulation test data for a hot climate with ideal humidity (temperature from 41 to 28 °C and 60% humidity).

**Fig. 16:**
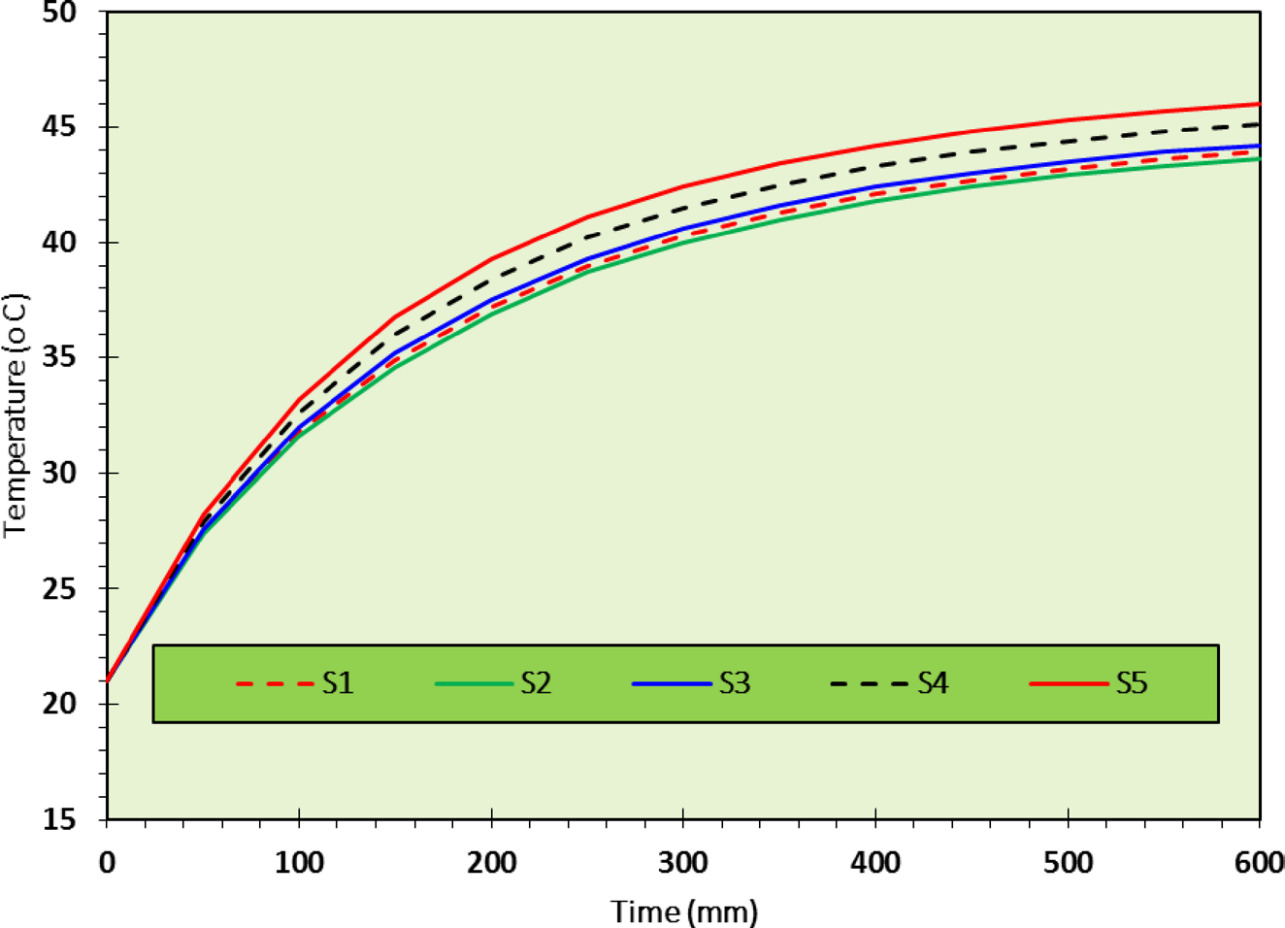
Thermal insulation test data for a hot climate with ideal humidity (temperature from 46 to 30 °C and 80% humidity).

As discussed above, the optimum mixture in the current study was CDW30-C2, so that a wall was constructed using this RE mixture, see Fig. 13. The thermal testing setup involved a controlled environment climate chamber maintaining precise temperature and humidity conditions (± 0.3 °C), with tests conducted at 40%, 60%, and 80% RH to represent dry, semi-dry/semi-humid, and humid conditions, respectively. Specimens were conditioned for 24 h to stabilize moisture content, and five digital temperature sensors (± 0.5 °C accuracy) were placed on the outer surface, inside the hollow core, and at the midpoint depth, sealed with ceramic fiber blankets to minimize heat exchange. The resulting thermal profiles presented in Figs. 14, 15, 16, show temperature changes over time for the CDW30-C2 wall under different humidity levels.

#### Hot climate with 40% relative humidity

Under 40% RH scenario, representing a dry hot climate, the thermal profile in Fig. 14 shows the temperature peaking at approximately 40 °C and stabilizing around 30 °C over 600 min. The TL, calculated as the delay between the external peak temperature (41 °C) and the internal peak (40°C), is approximately 50 min, indicating efficient heat transfer moderation. The decrement factor, derived as the ratio of internal temperature fluctuation (40.3 °C to 27 °C) to external fluctuation (41 °C to 27 °C), is about 0.95, suggesting minimal dampening of thermal amplitude due to low moisture content. This performance aligns with previous research findings^3,39,42^, who noted that low-humidity conditions enhance thermal inertia in dense earth materials. The peak internal temperature of 40 °C and rapid stabilization reflect the CDW30-C2 mixture’s effective insulation, attributed to the mechanical interlocking of CDW particles and the pozzolanic binding from 2% CaO, which reduces pore connectivity.

#### Hot climate with 60% relative humidity

At 60% RH scenario, simulating semi-dry to semi-humid conditions, Fig. 15 indicates a peak temperature of about 42 °C, with stabilization near 32 °C. The TL extends to around 70 min, reflecting a slower heat wave propagation due to increased moisture retention, consistent with^3,43^, who highlighted moisture’s role in enhancing thermal mass. The decrement factor, calculated from the internal fluctuation (40.6°C to 28 °C) against the external (42 °C to 28 °C), is approximately 0.90, showing improved dampening compared to 40% RH. The peak temperature rises to 42 °C, suggesting that moderate humidity slightly elevates heat retention, likely due to water’s thermal conductivity within the matrix. This demonstrates the mixture’s adaptability, though it indicates a need to manage moisture levels to optimize insulation.

#### Hot climate with 80% relative humidity

In the 80% RH scenario, representing humid conditions, Fig. 16 shows the highest peak temperature at approximately 45 °C, stabilizing around 35°C. The TL increases to about 90 min, the longest observed, due to significant moisture-induced thermal mass, as noted by previous work^3,43,44^. The decrement factor, based on the internal fluctuation (43.6 °C to 30 °C) versus the external (46 °C to 30 °C), is around 0.85, indicating the best thermal amplitude reduction among the tested conditions. However, the peak temperature increases to 45 °C and a slower cooling rate suggests reduced insulation efficiency, likely from excessive moisture filling voids and enhancing heat conduction. This aligns with the earlier observation that excessive CaO or moisture can create weak interfacial zones, emphasizing the importance of balancing CDW and CaO content for optimal thermal performance.

#### Comparative analysis

**Fig. 17:**
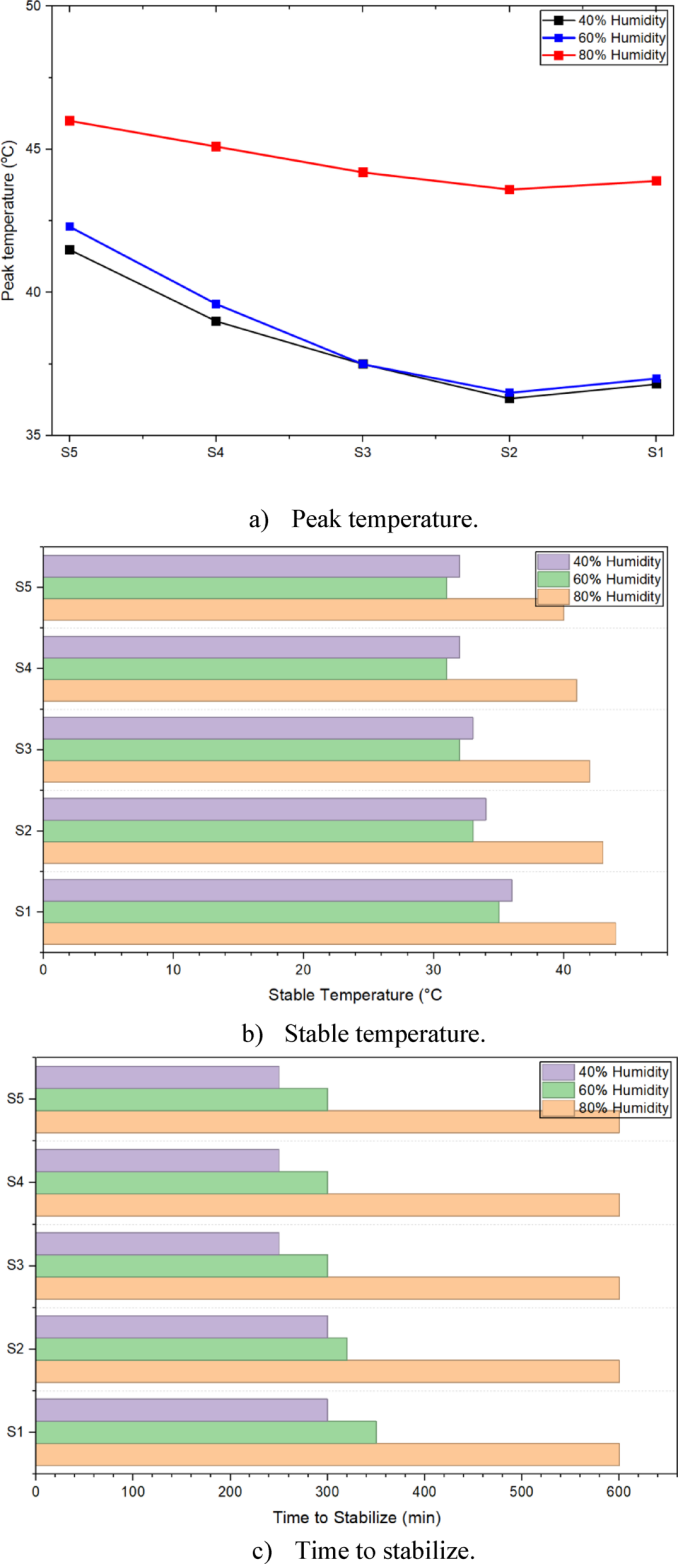
Peak temperature, stable temperature, and time to stabilize for the constructed RE-wall.

Compared to conventional materials, fired brick typically exhibits a time lag of 6–8 h and a decrement factor of 0.7–0.8 under similar conditions, while concrete shows a time lag of 5–7 h and a decrement factor of 0.6–0.7^3,45,46^. The CDW30-C2 mixture, with TL ranging from 50 to 90 min and decrement factors of 0.85–0.95, offers shorter lag times but comparable dampening, reflecting its lower thermal mass due to the soil base, see Fig. 17. However, its lower embodied energy (around 705.27 MJ/m^3^ from Fig. 12) and CO_2_ emissions (177.73 kg/m^3^) compared to brick (1000–2000 MJ/m^3^, 200–300 kg/m^3^) and concrete (800–1500 MJ/m^3^, 150–250 kg/m^3^) position it as a sustainable alternative. The results suggest that while the CDW30-C2 wall provides effective insulation in dry to moderately humid climates, its performance in high-humidity conditions could be enhanced with additional moisture-resistant additives or design adjustments.

## Conclusions

This study demonstrated the technical, thermal, and environmental feasibility of incorporating construction and demolition waste (CDW) and calcium oxide (CaO) as stabilizers in rammed earth (RE) construction. Microstructural and mineralogical changes were evaluated using scanning electron microscope (SEM) and X-ray diffraction (XRD) to explain the mechanisms of stabilization. Based on the derived results, the following conclusions are obtained.

1. Through systematic experimentation and characterization, the CDW30-C2 mix (30% CDW and 2% CaO) emerged as the optimal formulation, offering a balanced performance across mechanical, thermal, and sustainability criteria.
2. The addition of CDW enhanced compressive strength through enhanced particle interlocking and densification, while CaO introduced effective pozzolanic reactions, forming cementitious compounds that contributed to long-term strength development. The optimal mix achieved a 28-day unconfined compressive strength of 9.3 MPa—more than double the value of the un-stabilized reference soil.
3. The thermal behavior of the optimized CDW30-C2 mixture further highlighted its suitability for passive thermal regulation. It exhibited the lowest thermal conductivity (0.88 W/m·K), with a favorable decrement factor (0.85–0.95) and time lag (50–90 min) under variable humidity conditions, confirming the wall’s capacity to moderate indoor temperatures in hot climates. While its thermal inertia did not match that of fired brick or conventional concrete, the mix provided sufficient insulation for arid and semi-arid applications, and its performance under humid conditions suggests opportunities for further optimization through admixture design.
4. The CDW30-C2 mix had great environmental and economic benefits. Its CO_2_ emissions (177.73 kg/m^3^), embodied energy (705.27 MJ/m^3^) were significantly lower compared to traditional materials such as fired brick and concrete or similar types of construction materials, which fulfill global objectives in decreasing the environmental footprint of construction materials. The cradle-to-gate life cycle assessment concluded that a moderate CDW content, including low CaO, offers overall environmental burden reduction and a high strength-to-cost and strength-to-emissions ratio.

Overall, these results validate that CDW and CaO can coexist in RE systems for improved mechanical performance and thermal comfort, while sustaining sustainability. This would benefit circular economy efforts by giving waste materials value, whilst reducing reliance on carbon-intensive conventional binders. Future research could involve the investigation of long-term durability subjected to hydrothermal cycling in the field with wall prototypes to be used in the real world, and also with hydrophobic additives to make the systems applicable to more humid environments.

## Data Availability

All data generated or analyzed in this study are included in this manuscript.
